# Application of a Loop-Type Laboratory Biofilm Reactor to the Evaluation of Biofilm for Some Metallic Materials and Polymers such as Urinary Stents and Catheters

**DOI:** 10.3390/ma9100824

**Published:** 2016-10-11

**Authors:** Hideyuki Kanematsu, Hikonaru Kudara, Shun Kanesaki, Takeshi Kogo, Hajime Ikegai, Akiko Ogawa, Nobumitsu Hirai

**Affiliations:** 1 Department of Materials Science and Engineering, National Institute of Technology, Suzuka College, Suzuka Mie 510-0294, Japan; h28b03@ed.cc.suzuka-ct.ac.jp (H.K.); h28b02@ed.cc.suzuka-ct.ac.jp (S.K.); kogo@mse.suzuka-ct.ac.jp (T.K.); 2 Department of Chemistry and Biochemistry, National Institute of Technology, Suzuka College, Suzuka Mie 510-0294, Japan; ikigai@chem.suzuka-ct.ac.jp (H.I.); ogawa@chem.suzuka-ct.ac.jp (A.O.); hirai@chem.suzuka-ct.ac.jp (N.H.)

**Keywords:** biofilm, loop-type laboratory biofilm reactor, Raman spectroscopy

## Abstract

A laboratory biofilm reactor (LBR) was modified to a new loop-type closed system in order to evaluate novel stents and catheter materials using 3D optical microscopy and Raman spectroscopy. Two metallic specimens, pure nickel and cupronickel (80% Cu-20% Ni), along with two polymers, silicone and polyurethane, were chosen as examples to ratify the system. Each set of specimens was assigned to the LBR using either tap water or an NB (Nutrient broth based on peptone from animal foods and beef extract mainly)—cultured solution with *E-coli* formed over 48–72 h. The specimens were then analyzed using Raman Spectroscopy. 3D optical microscopy was employed to corroborate the Raman Spectroscopy results for only the metallic specimens since the inherent roughness of the polymer specimens made such measurements difficult. The findings suggest that the closed loop-type LBR together with Raman spectroscopy analysis is a useful method for evaluating biomaterials as a potential urinary system.

## 1. Introduction

Biomaterials have been investigated and developed with regards to mechanical/material engineering functionalities [1] leading to the structural advancement of many materials. The biological compatibilities of potential biomaterials need to be investigated concurrently, in particular, their material performance characteristics in terms of controlling infections. Such factors have nowadays become important issues [2].

The urinary tract (UT) system investigated in this study, for example, has issues relating to infection due to stent placements indwelling catheters [3,4,5,6,7,8,9,10,11,12]. The stent is used for patients suffering from ureter blockages due to urinary stones, and in those cases when general anesthesia for catheter-based therapy or repeated ultrasound disintegration cannot be used. However, the urinary stent has to be replaced after a certain time since it becomes tainted and could be the catalysis for a secondary source of infectious diseases. Furthermore, the replacement procedure itself may cause patient further burden and anguish. Therefore, it is meaningful to develop novel materials with anti-biofilm characteristics. Firstly, it is necessary that methods to ascertain biofilm formation be established. Even though the biological assessment of biofilms has been investigated often, a standard evaluation system has been lacking from the viewpoint of materials science.

This research investigates suitable analytical methods to study biofilm formation on materials, not from a biological viewpoint, but from a materials science and engineering perspective. Any such system combines both the biofilm formation process and its evaluation. The authors have designed and produced a loop-type circulation laboratory biofilm reactor (LBR) [13,14,15,16,17,18,19,20,21,22], where biofilms have been evaluated by crystal violet staining, 3D optical microscopy, SEM-EDX (lower vacuum condition), Raman Spectroscopy and FTIR-ATR. The study pays particular attention to the production and evaluation of biofilms pertaining to the urinary system, and potential materials for catheters and stents. Initially, the LBR system was modified to work outside of a human body and investigations were undertaken involving the artificial and accelerated production of biofilm, including their evaluation by analytical methods.

## 2. Experimental

### 2.1. Specimens and LBR

Two metallic specimens (pure nickel, and cupronickel (80% Cu-20% Ni) sheets) and two polymeric materials (silicone and polyurethane sheets) were used. The original aim was to develop nickel-copper coatings to serve as a biomaterial substrate. However, metallic sheets were substituted for the studies. Even though there are many commercially available biomaterial polymers, the polymeric specimens employed in this study are often used as biomaterials. The heat-resistant polymers were autoclaved at 125 °C for 20 min. Having two kinds of specimens as examples for this study (total four specimens: two metallic and two polymeric materials), the focus was to establish an appropriate evaluation system comprising both the production and the measurement on biofilm materials, thereby allowing fundamental data to be obtained concurrently. Prior to the trials, the specimens were cut into sheets 1 cm × 2 cm (the thickness ranged from 0.1 mm to 1 mm). They were sandpapered using #1000 abrasive papers (JIS R-6010) and cleaned by immersing into ethanol solution (99.5%) for a couple of minutes.

**Figure 1:**
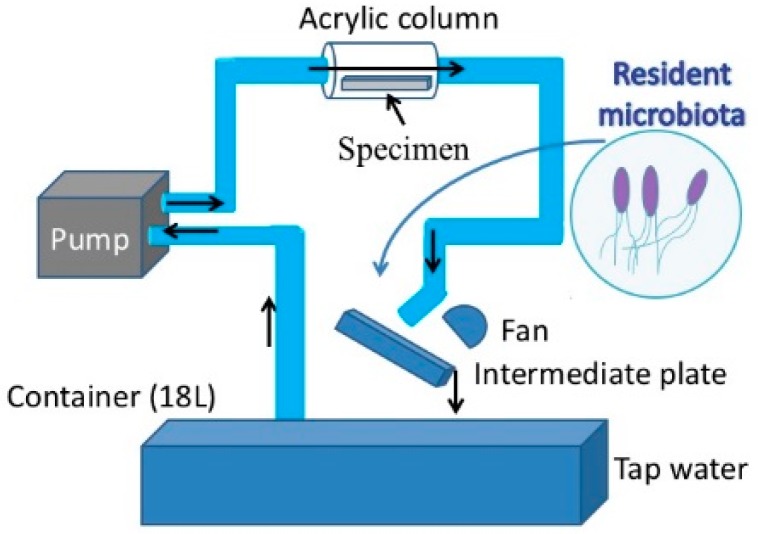
Previous laboratory biofilm reactor (LBR) for our studies.

**Figure 2:**
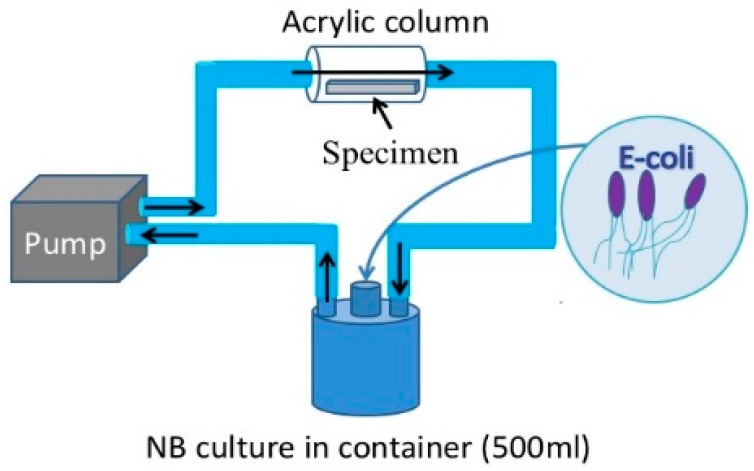
The current LBR for this investigation.

For the purpose of this study a new loop-type LBR was developed. Lebeaux et al. [23] classified LBRs used for in vitro biofilm research into three types of systems, namely, static, open and microcosmos. Our LBR is an open type LBR system under this classification. Such classification is important as it clarifies the interaction with the environment. Our initial LBR developed in previous studies is shown schematically in Figure 1 [13,14,15,16,17,18,19,20,21,22]. Water mixed with resident microbiota in the atmosphere is circulated around the system, allowing a biofilm to form on the specimens located in the acrylic pipe. The biofilm specimens had an unknown number of bacteria. Since the aim was to control and determine certain bacteria in urinary systems, the system was modified and a new LBR system produced as shown in Figure 2, described here as a closed-loop system.

Two types of solutions circulating in the loop were tested as part of this study. One solution was tap water taken from Suzuka city, where all the authors live and work. The bacteria in the water was unspecified collected germs. The other solution trialed was a liquid culture comprising *Escherichia coli (E-coli)*. In the case of tap water, a 500 mL glass container with three mouths was filled with water. An acrylic pipe was then connected with silicon tubes to the container through a peristaltic pump (TP-1973R, AS ONE Co. Ltd., Osaka, Japan). The tap water was pumped from the container to the acrylic pipe where specimens were held in place using a special jig. The solution circulated around the pipe and container. The process continued for 48 h (2 days)–72 h (3 days) at a flow rate of 5 mL/min. The test period was 48 h for *E-coli* and to 72 h for resident microbiota. As for the culture media process, *E-coli* (K12G6) was incubated for 24 h so that the bacteria numbered around 10^9^ cfu/mL. Then 0.1 mL of the culture was diluted with 0.9% NaCl solution tenfold (the number of bacteria: 10^7^ cfu/mL at this point). 12 mL glucose (10%) was then added to the diluted culture solution and was added into nutrient broth (290 mL). Finally, the culture solution was prepared so that it contained 3.3 × 10^4^ cfu/mL bacteria and 0.4% glucose. The container, acrylic pipes with specimens and silicon tubes were autoclaved at 125 °C for 20 min prior to the tests. Experiments using the cultured solution were performed in the same way as the tap water investigations. However, one of the glass container’s mouths was closed by a filter which allowed only air without bacteria, while other two mouths were used for the inlet and outlet for solutions.

### 2.2. Evaluation of Biofilms

Three metallic materials were evaluated by an optical microscope equipped with a 3D display system (VW-9000, Keyence Co., Osaka, Japan) and Raman spectroscopy (NRS-3100, JASCO Co., Tokyo, Japan). The specimens were removed from the acrylic pipe after immersion and immediately examined under an optical microscope. The microscope stage was shifted around the focal point of each specimen and 3D images obtained. The PC software of the optical scope assigned a color to the specimen’s surface according to changes in height. The resulting sea-island patterns were obtained only when biofilms formed on the specimens. The measurements were only possible on metallic materials that were immersed in tap water. For the polymeric materials, the inherent surface profiles were very rough, prohibiting the 3D optical microscope to differentiate between the surfaces before and after immersion.

Specimens taken out of the LBR were freeze-dried in the following way. A dewatering process was carried out using ethanol and t-butyl alcohol. First of all, the specimens were immersed into ethanol solutions to replace the water in biofilms. Various ethanol concentrations of 30%, 50%, 60%, 70%, 80%, 90%, 95%, 98% and 99.5% were prepared, after which each specimen was placed in a small pill case and ethanol gradually added using a dropper. The specimens were then immersed into different ethanol concentration for 15 min and drawn and discharged by the dropper. The substitution process between water and ethanol was repeated for all 9 different ethanol solutions, in order of increasing concentration. After substitution, it was replaced by t-butyl alcohol, using the mixed solutions. The ratio of ethanol to t-butyl alcohol was changed in the order of 70/30, 50/50 and 30/70, so that the ethanol was gradually exchanged with t-butyl alcohol. Finally, the specimens were immersed into 100% butyl alcohol and placed in a freezer for 30 min. The frozen specimens were placed in a desiccator which was evacuated until the t-butyl alcohol completely evaporated. The specimens were observed by Raman spectroscopy and optical microscopy. For Raman measurements, the specimen surface was examined using an optical microscope and precipitates and the periphery specifically irradiated to obtain the Raman peak shifts. The Raman peak shifts of the results obtained in this study were qualitatively compared with those in the literature.

## 3. Results and Discussion

### 3.1. Metallic Specimens

**Figure 3:**
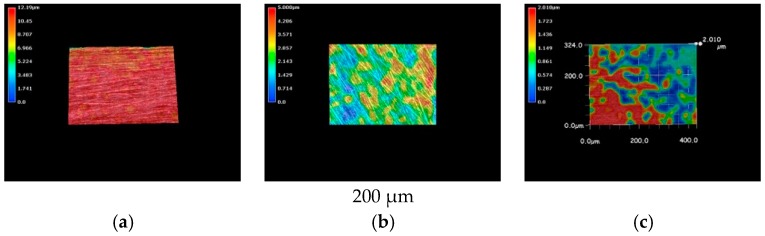
The 3D images by the optical microscopy for pure nickel specimens. (**a**) Before immersion; (**b**) resident microbiota; (**c**) *E-coli*.

The metallic specimens were immersed into the LBR filled with tap water and also immersed into the LBR filled with culture solution of *E-coli* for 72 h. The 3D optical images of pure nickel specimens are shown in Figure 3. The red color corresponds to areas with the greatest heights, whereas the blue depicts low-height areas. Before immersion, the photo shows the specimen to have a relatively homogenous profile (Figure 3a) However, specimens immersed in either the LBR with resident microbiota (Figure 3b) or with *E-coli* (Figure 3c) show sea-island patterns. *E-coli* shows greater height variations than compared with the result of resident microbiota.

**Figure 4:**
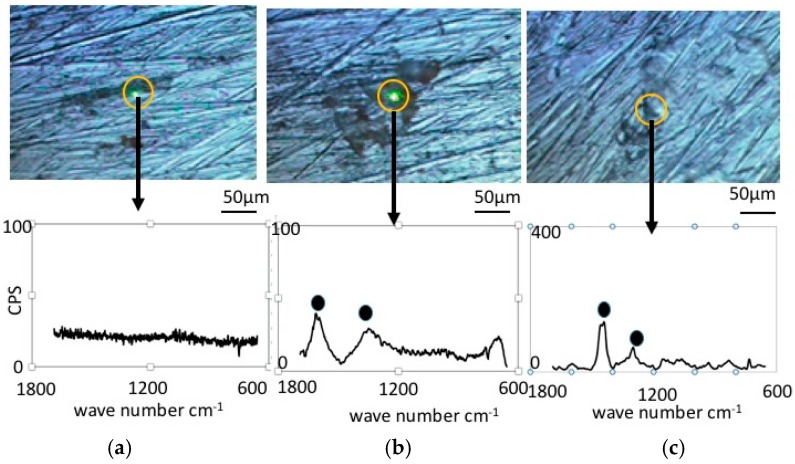
Raman shift peaks and the optical microscopic images for pure nickel specimens. (**a**) Before immersion; (**b**) resident microbiota; (**c**) *E-coli*.

Figure 4 shows Raman Spectroscopy results obtained for the pure nickel specimens. Before immersion (Figure 4a), the specimens did not exhibit any significant peaks, which is expected since metallic bonding typically does not show Raman shifts. However, the specimen immersed in the LBR with resident microbiota (Figure 4b) showed peaks at 1368 cm^−1^ and 1600 cm^−1^. We postulate that these peaks are derived from the biofilm itself or from related organic compounds. It may include any organic compounds that attach themselves to the sticky surface of the specimens due to the formation of a biofilm. Figure 4c shows the results of Raman spectroscopy in the case of *E-coli*. All peaks were considered to derive from the biofilm according to a comparison with data from literature [24,25,26,27,28,29,30]. Concretely speaking, we presume that the peak at 1368 cm^−1^ corresponds to lipids and the peak at 1600 cm^−1^ to Amid I.

**Figure 5:**
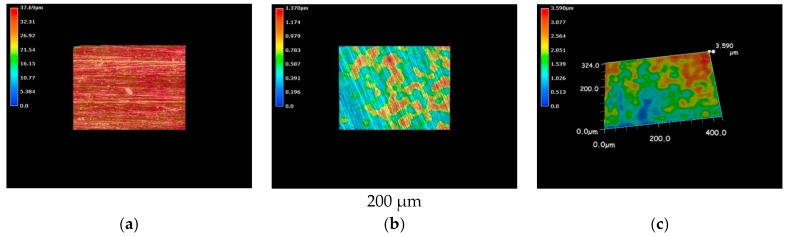
The 3D images by the optical microscopy for cupronickel specimens. (**a**) Before immersion; (**b**) resident microbiota; (**c**) *E-coli*.

The results from optical microscopic observations of cupronickel specimens are shown in Figure 5. Like in the case of pure nickel, cupronickel also exhibits sea-island patterns after immersion into LBR with both resident microbiota and *E-coli* (Figure 5b,c), while the specimen before immersion showed a homogeneous surface profile (Figure 5a). The tendency could be explained by the same reasons as described above for pure nickel specimens. The extent of the sea-island pattern for *E-coli* was greater than that for resident microbiota, like in the case of pure nickel. However, the pattern was not so clear for cupronickel specimens compared with the results for nickel specimens. Even though microscopic observations did not provide quantitative data, the results suggest that copper in the cupronickel substrate worked to control the bacterial growth on the specimens’ surfaces, leading to the inhibition of biofilm formation to some extent.

**Figure 6:**
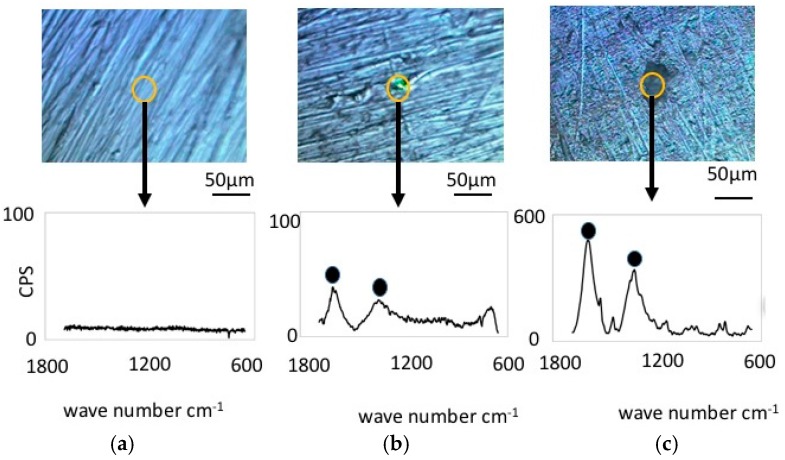
Raman shift peaks and the optical microscopic images for cupronickel specimens. (**a**) Before immersion; (**b**) resident microbiota; (**c**) *E-coli*.

Figure 6 shows the results from Raman spectroscopy of the cupronickel specimens. Before immersion, the specimens exhibited no Raman shifts, similar to the case of nickel specimens. However, the specimens immersed in LBR and exposed to bacteria exhibited some Raman peak shifts for both the resident microbiota and *E-coli*. Significant Raman peaks were observed at 1060 cm^−1^, 1368 cm^−1^ and 1600 cm^−1^ for the specimen exposed to resident microbiota, while the significant peaks for the specimen exposed to *E-coli* were observed at 1300 cm^−1^ and 1450 cm^−1^. In both cases the peaks are believed to derive from the biofilm, which means that the EPS or the attached carbon compounds are due to the stickiness of the surfaces. The former should be considered polymers in biofilms (EPS) as well as e-DNA and bacteria themselves. All of them could be components of biofilms. On the other hand, the latter might be just the organic compound existing in the liquid phase outside of biofilms. They might attach to materials surfaces due to their stickiness caused by EPS from biofilms. The latter could be also considered biofilm derived carbon compounds in the broad sense of the term [24,25,26,27,28,29,30]. From the viewpoint of industrial applications, matter arriving internally and externally to the biofilms would make an even contribution to dirt and surface contamination. Therefore, in this paper, we do not term both peaks as “bacteria derived peaks”, but “biofilm derived peaks”.

Comparing the Raman spectra shown in Figure 4 and Figure 6, one can see that the peaks are not similar even though the bacterial flora and species were the same. The Raman peaks in the case of *E-coli* were generally higher than those in resident microbiota. These findings suggest that the combination of substrate materials and bacterial species would fix the organic compounds and the corresponding Raman shifts.

The series of experiments described above suggest that Raman spectroscopy could be a useful tool to evaluate biofilm formation on materials surfaces.

### 3.2. Polymer Specimens

Two types of polymers were chosen to establish what kind of evaluation methods would be appropriate from the viewpoint of materials science. However, the study is based from the viewpoint of biomaterials and also their heat resistance, as described above. Studies focused on Raman spectroscopy evaluations for specimens immersed in the LBR with *E-coli*.

**Figure 7:**
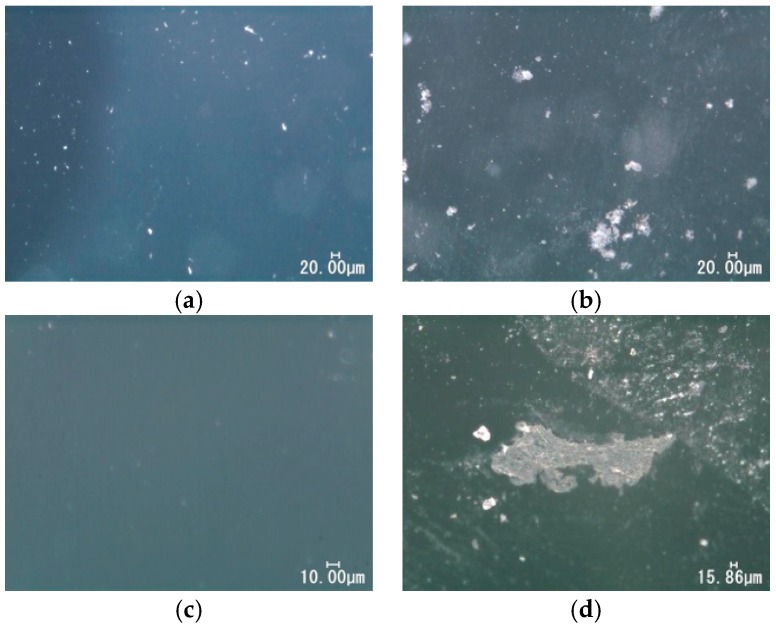
Optical microscopic images for polymeric specimens. (**a**) Silicon before immersion; (**b**) silicon after immersion; (**c**) polyurethane before immersion; (**d**) polyurethane after immersion.

Figure 7 shows the usual optical microscopic images for silicone and polyurethane before and after the immersion into the LBR. For both specimens, precipitates were observed after immersion, even though those could be rarely seen on the surfaces before the immersion.

**Figure 8:**
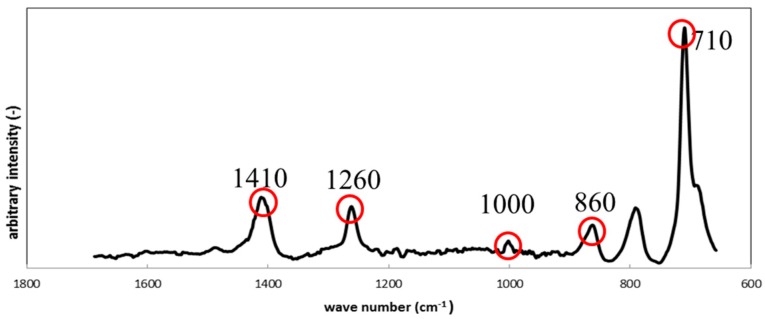
Raman shifts for the silicone specimen before immersion.

Figure 8 shows the results from Raman Spectroscopy for the silicone specimens before the immersion. The characteristic Raman shifts for the specimens were observed at 710 cm^−1^, 860 cm^−1^, 1000 cm^−1^, 1260 cm^−1^ and 1410 cm^−1^, and corresponded to silicone apart from a peak at 790 cm^−1^, which could not be identified. We assume that the unidentified peak could be attributed to an unknown component(s) mixed during the preparation procedure.

**Figure 9:**
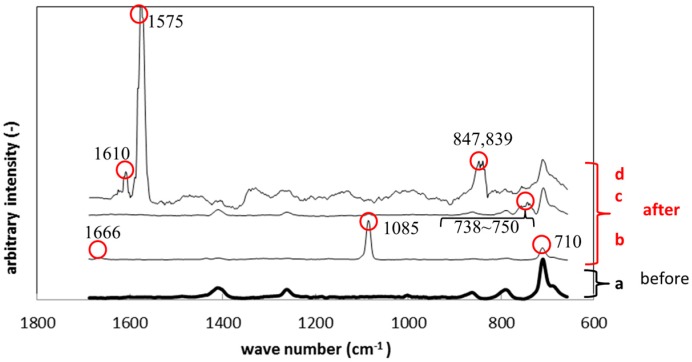
Raman shifts for the silicone specimens before (**a**) and after immersion (**b**–**d**).

After immersion, the specimen showed different peaks from those before immersion. Figure 9 shows Raman peak shift of silicone specimens before and after the immersion. Figure 9a shows the result before immersion, while Figure 9b–d show the results after immersion. The colored circles identify the peaks which did not appear before immersion. The peaks differed from place to place on the biofilm surface since the biofilm was not homogenously formed. The peaks observed in Figure 9b (710, 1085, 1666 cm^−1^) correspond to calcium carbonate, which is contained in the polymer specimens as an additive. The peaks between 738 to 750 cm^−1^ belong to polysaccharide, which was derived from exopolymeric substance (EPS). The peaks at 839 and 847 cm^−1^ in Figure 9d also belong to polysaccharide in EPS. The highest peak at 1575 cm^−1^ in Figure 9d also corresponded to that of gluconic acid derived from the NB culture.

**Figure 10:**
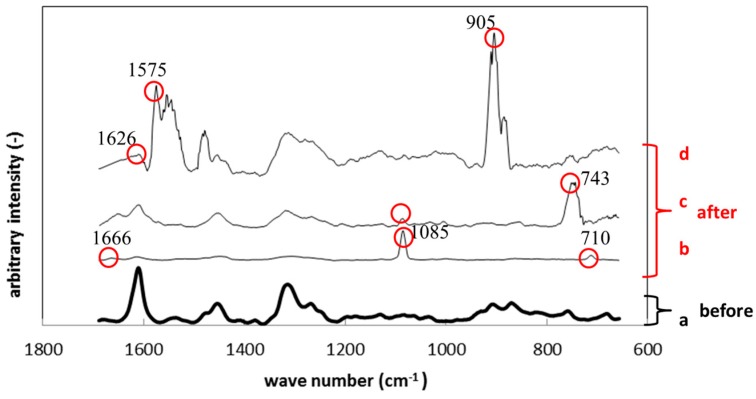
Raman shifts for the polyurethane specimens before (**a**) and after immersion (**b**–**d**).

Figure 10 shows Raman spectroscopy results of polyurethane specimens before and after the immersion. Just like Figure 9, Figure 10a corresponds to the result before the immersion and Figure 10b–d to those after the immersion. The circled peaks appeared after the immersion. The peaks at 710 cm^−1^, 1085 cm^−1^ and 1666 cm^−1^ in Figure 10b correspond to the Raman shift of calcium carbonate, like the results in Figure 9b. The Raman peak at 743 cm^−1^ in Figure 10c was considered polysaccharide in EPS. The large peaks around 1570 cm^−1^ in Figure 10d belong to gluconic acid, while the peak at 905 cm^−1^ corresponds to polysaccharide in EPS.

All this data suggests that the loop-type LBR system and Raman spectroscopy could be used for the evaluation of biofilms formed on stent materials.

## 4. Conclusions

A new LBR system was devised and a biofilm evaluation process of potential stent materials used in urinary systems investigated using optical microscopy with 3D display function and Raman spectroscopy. The key findings derived from this study are highlighted.

(1) The closed loop-type LBR devised for this study could be used as a new accelerated LBR for the evaluation of materials found in urinary systems. Biofilms formed in short periods of time (48–72 h) using either residential microbiota or *E-coli*.
(2) 3D optical microscopy proved to analyze metallic materials as their as-received surface profiles were relatively smooth. The method employed was semi-quantitative for biofilm evaluation.
(3) The 3D surface profiles suggest that the single-strain system highlighted the biofilm more clearly than the mixed resident microbiota, and that copper’s antimicrobial effect could control biofilm formation.
(4) Raman spectroscopy proved useful for both metallic and polymer materials when combined with the loop-type LBR. A qualitative analysis of the Raman findings revealed that the EPS in the biofilm containing the carbon compounds attached to the specimens’ surfaces due to the stickiness of the biofilm.
